# Age-Dependent Progression of Neurological Involvement in PRPP Deficiency: Insights from a Four-Generation Family and Systematic Review

**DOI:** 10.3390/biom16081164

**Published:** 2026-08-11

**Authors:** Bartosz Rodziewicz, Mikołaj Kacperski, Kacper Kisiński, Marta Zawadzka, Anna Kalicka, Agnieszka Sawicka, Beata Lipska-Ziętkiewicz, Maria Mazurkiewicz-Bełdzińska

**Affiliations:** 1 Department of Developmental Neurology, Medical University of Gdansk, 80-952 Gdansk, Poland; m.kacperski@gumed.edu.pl (M.K.); k.kisinski@gumed.edu.pl (K.K.); agnieszka.sawicka@gumed.edu.pl (A.S.); maria.mazurkiewicz-beldzinska@gumed.edu.pl (M.M.-B.); 2 Clinical Genetics Unit, Rare Diseases Centre, University Clinical Center, 80-210 Gdansk, Poland; akalicka@uck.gda.pl (A.K.); beata.lipska-zietkiewicz@gumed.edu.pl (B.L.-Z.); 3 Clinical Genetics Unit, Department of Biology and Medical Genetics, Medical University of Gdansk, 80-210 Gdansk, Poland

**Keywords:** *PRPS1*, diagnostic latency, neurometabolic disorders, sensorineural hearing loss

## Abstract

Loss-of-function (LoF) variants in the *PRPS1* gene, encoding the phosphoribosyl pyrophosphate (PRPP) synthetase 1 enzyme, cause rare neurometabolic disorders historically viewed as discrete entities: nonsyndromic deafness (DFNX1), Charcot–Marie–Tooth disease type X5 (CMTX5), and Arts syndrome. A major clinical challenge is the temporal dissociation between early auditory failure and subsequent neurodegeneration, causing fragmented diagnostics. We systematically quantified this diagnostic latency and reconceptualized the disease spectrum through a molecular lens. A PRISMA-compliant systematic review identified 19 patients with genetically confirmed *PRPS1* LoF variants, including our index case (c.362C>G) presenting a 15-year diagnostic delay. Kaplan–Meier analysis revealed sensorineural hearing loss manifested acutely (median 0 years; 95% CI: 0–1). In contrast, neurological deficits demonstrated a prolonged latency (median 3 years; 95% CI: 1–8), followed by ophthalmological signs (median 11.5 years). The median symptomatic delay was 3 years (range up to 19). We posit that this temporal dissociation reflects differential tissue vulnerability to intracellular ATP/GTP and NAD+ depletion caused by the primary enzymatic defect. Ultimately, DFNX1, CMTX5, and Arts syndrome represent a continuous *PRPS1*-related neurometabolic spectrum. Because targeted metabolic interventions (such as S-adenosylmethionine or nicotinamide riboside) have limited efficacy on advanced structural nerve damage, recognizing this early diagnostic window to initiate biochemical rescue prior to irreversible axonal degeneration is critical.

## 1. Introduction

Hereditary motor and sensory neuropathies (HMSN), broadly classified as Charcot–Marie–Tooth (CMT) disease, constitute the most common group of inherited neuromuscular disorders, with a prevalence of approximately 1 in 2500 [1,2]. Although the classical phenotype involves distal muscle atrophy and sensory deficits [3], the identification of several genetic variants, notably the X-linked subtypes, has expanded the clinical spectrum to include central nervous system involvement [4]. Within this genetic landscape, the *PRPS1* gene (phosphoribosyl pyrophosphate synthetase 1) presents a specific diagnostic challenge [5].

*PRPS1* encodes phosphoribosylpyrophosphate (PRPP) synthetase, an enzyme essential for nucleotide biosynthesis and purine metabolism [6]. Reduced enzymatic activity due to underlying loss-of-function (LoF) gene variants results in a series of interrelated phenotypes [7], ranging from nonsyndromic sensorineural hearing loss (DFNX1; ORPHA:90625 [8]) [9] through Charcot–Marie–Tooth disease type X5 (CMTX5; ORPHA:99014 [8]) characterized by hearing loss, neuropathy, and optic atrophy [10] to the severe Arts syndrome (ORPHA:1187 [8]), which includes ataxia, early-onset hypotonia, delayed motor development, hearing loss, optic atrophy, intellectual disability, and susceptibility to infections [11]. Since these conditions share a common pathophysiology linked to enzyme deficiency, the clinical boundaries are often indistinct [12]. Likewise, PRPP synthetase superactivity due to gain-of-function (GoF) *PRPS1* gene variants results in purine overproduction, causing early-onset hyperuricemia and hyperuricosuria, and clinically manifesting with urolithiasis, gout, and neurodevelopmental anomalies consisting of variable combinations of sensorineural hearing loss, hypotonia, and ataxia (ORPHA: 411536; 411543 [8]).

A major diagnostic challenge in PRPP synthetase deficiency is the time gap between early congenital hearing loss and later-onset neurological or visual impairments [7,13]. To overcome this fragmented presentation, early diagnosis relies on a high index of clinical suspicion and detailed intergenerational family history. Furthermore, the disease severity and phenotypic variability are directly driven by the specific molecular features of the *PRPS1* variant, particularly its structural location and impact on hexamer stability. While some variants lead to stable, isolated sensory impairments, others cause progressive neurodegeneration. Consequently, family case studies are essential for understanding the natural history and variable penetrance of the disease. Recognizing this continuum early provides a crucial window to initiate emerging, disease-modifying metabolic therapies before irreversible structural damage occurs.

To illustrate this diagnostic challenge, we present a four-generation family carrying a pathogenic *PRPS1* variant. By integrating our clinical findings with a systematic literature review, we map the age-dependent progression of the disease from acute, isolated hearing loss in infancy to delayed, severe neurodegeneration and vision loss. Our objective is to raise clinical awareness and accelerate the diagnostic process for children with congenital deafness who are at risk for progressive neuropathy.

Furthermore, we link these clinical observations to the molecular architecture of the PRPP synthetase 1 enzyme. We explore how specific structural defects reduce enzymatic activity and propose how this deficiency may trigger purinosome dysregulation and intracellular NAD+ depletion, the underlying mechanisms hypothesized to drive this continuous disease spectrum.

## 2. Case Presentation

The index case is a Caucasian male patient with a 15-year diagnostic latency between the onset of sensorineural hearing loss and neurological deterioration. At two months of age, absent responses to loud sounds prompted evaluation, and by seven months, bilateral profound sensorineural hearing loss was confirmed. Early motor development was initially normal, with independent walking achieved at twelve months. Following cochlear implantation at 22 months, gradual speech development occurred. He later attended a vocational school for the hearing-impaired; cognitive assessment indicated average intellectual functioning.

At age 16, he was admitted to the Department of Developmental Neurology (University Clinical Centre, Gdańsk) for progressive gait disturbance. Past medical history (as reported by the mother) noted early-childhood clumsiness and falls, initially attributed to hearing impairment. Over the preceding two years, motor coordination markedly deteriorated, featuring frequent falls, inability to squat or rise from the floor unaided, fine motor difficulties, and physical activity intolerance.

Neurological examination revealed reduced lower extremity muscle strength, absent deep tendon reflexes in the legs, pes cavus requiring orthopedic insoles, and quadriceps atrophy. Sensation remained intact. At age 16, he was subjected to immunological screening following an adverse drug reaction that showed no evidence of primary immunodeficiency. Ophthalmological involvement was first characterized by abnormal visual evoked potentials (VEPs) at age 16, with subsequent optical coherence tomography (OCT) at age 19 demonstrating features of bilateral optic neuropathy with secondary extramacular maculopathy.

**Figure 1:**
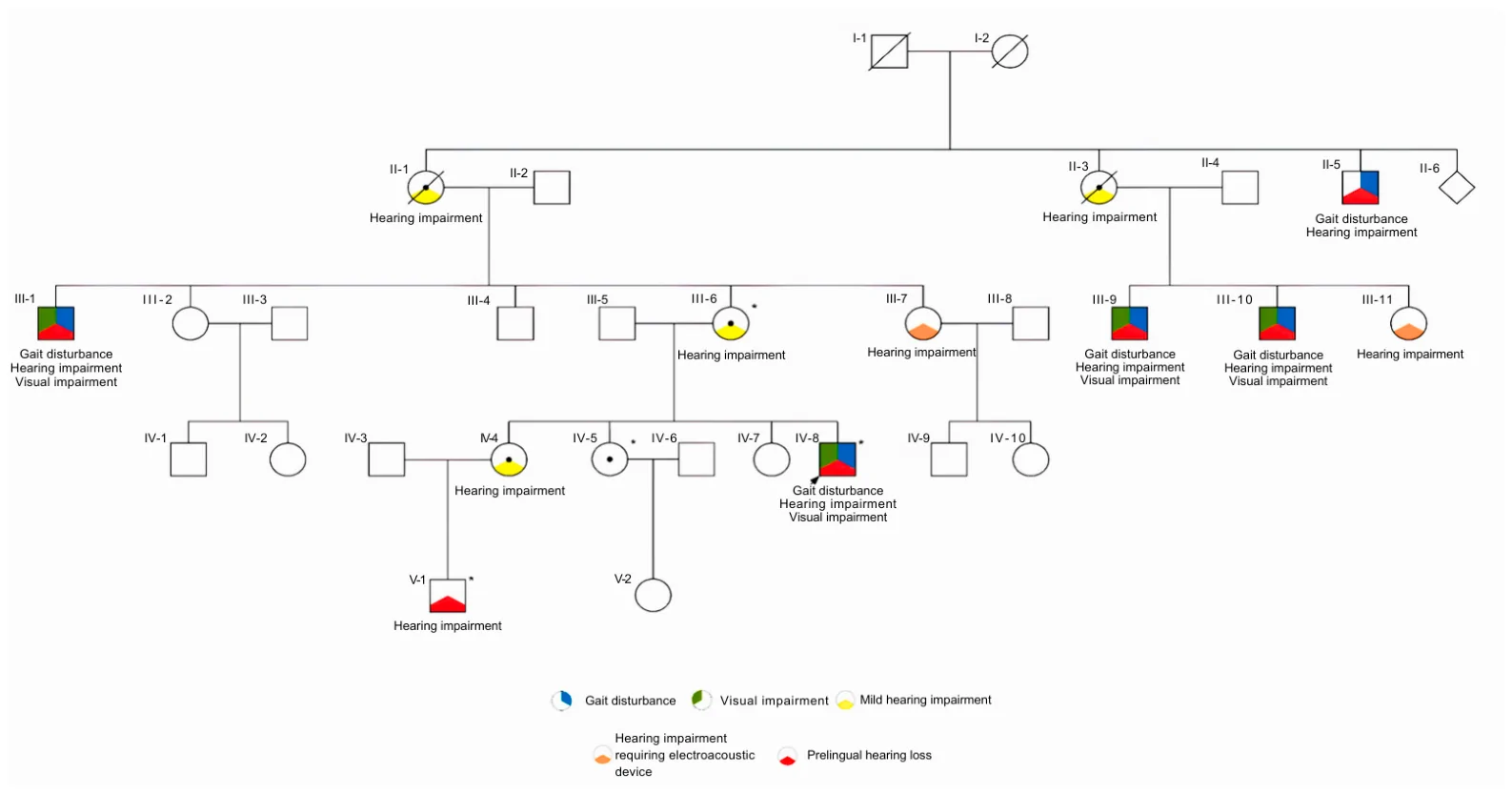
Four-generation pedigree of the reported family; index case indicated by an arrow. The diagram was generated using PedigreeTool version 0.5.2 Beta [16]. The asterisk (*) indicates individuals who underwent molecular genetic testing.

Family history revealed five maternal male relatives with deafness, vision impairment, and progressive gait deterioration, strongly suggesting an X-linked inheritance pattern and guiding further diagnostics (Figure 1). Genetic testing (a large virtual NGS panel performed on exome-derived sequences) identified a known hemizygous pathogenic variant in *PRPS1* (NM_002764.4: c.[362C>G];[0]), previously described in a young Korean individual with a clinical diagnosis of CMTX5 [14]. This missense variant (p.Ala121Gly), listed in ClinVar (ID 100767) [15], affects a highly conserved residue and is absent from population databases and currently lacks functional characterization.

Following genetic confirmation, oral S-adenosylmethionine (SAM, 250 mg/day) was initiated. Despite the implementation of metabolic treatment, the clinical prognosis for this patient remains unfavorable.

The patient provided informed consent for the publication of these diagnostic and therapeutic measures, contributing a valuable perspective on the illness course.

Detailed phenotyping revealed additional affected relatives, including females (Figure 1). Five male maternal relatives were affected with peripheral neuropathy and hearing loss; three of them also had visual impairment, including one individual with complete vision loss. Individual III-1 was diagnosed with hearing loss at 2 years of age, initially attributed to gentamicin treatment for bacterial meningitis. Significant lower-limb weakness developed at approximately 14 years of age, and he became wheelchair-dependent at 60 years. He also exhibited visual impairment while retaining functional vision. Individual III-9 was diagnosed with hearing loss during the first year of life, developed complete vision loss at 47 years of age, and lost ambulation at 55 years; the age at onset of gait impairment was unavailable. Individual III-10 was diagnosed with hearing loss during the first year of life and developed significant lower-limb weakness at 15 years of age, when he was diagnosed with peripheral neuropathy. He became wheelchair-dependent at approximately 53 years of age and has visual impairment with preserved vision.

Among female relatives, six were affected by hearing impairment of variable severity, including one confirmed heterozygous carrier of the variant and four others, who were obligate carriers of the variant based on pedigree analysis. Two of the affected females (III-7, III-11) required electroacoustic hearing devices in the 1st–2nd decades of life. Individual II-3 developed a steppage gait at the age of 70. One female (IV-5), a confirmed carrier of the variant, was diagnosed with epilepsy at the age of 26.

## 3. Materials and Methods

### 3.1. Search Strategy and Protocol

This systematic literature review was conducted in accordance with the Preferred Reporting Items for Systematic Reviews and Meta-Analyses (PRISMA) guidelines (see File S1 for the completed checklist) [17]. A comprehensive search was performed across three major electronic databases: PubMed, Web of Science (Core Collection), and Scopus. The search aimed to identify all relevant published case reports, case series, and original articles describing patients with *PRPS1* gene defects and associated clinical phenotypes. Each database was searched from its inception to 1 March 2026, with no language restrictions applied. To ensure a highly sensitive search, the strategy utilized a combination of free-text words and database-specific subject headings. The search combined terms related to the *PRPS1* gene with keywords encompassing the core clinical manifestations of the disease spectrum, including auditory, neurological, and ophthalmological features (“deafness”, “hearing loss”, “neuropathy”, “ataxia”, “gait”, “optic atrophy”, and “retina”).

The review protocol was not prospectively registered, which we acknowledge as a methodological limitation. This was primarily due to the purely observational nature of the included case reports, which generally do not meet the eligibility criteria for major registries such as PROSPERO.

### 3.2. Eligibility Criteria

Inclusion criteria:
  (1) Individuals with a genetically confirmed pathogenic or likely pathogenic variant in the *PRPS1* gene, presenting with clinical phenotypes within the DFNX1, CMTX5, or Arts syndrome spectrum.
  (2) Availability of individual-level clinical data regarding the age at onset of both core symptoms (auditory and neurological).
  (3) Peer-reviewed original articles, case reports, or case series with full texts available.
Exclusion criteria:
  (1) Animal models, in vitro experiments, or functional studies lacking novel human clinical data.
  (2) Gain-of-function (GoF) variants causing PRPP synthetase superactivity (purine overproduction disorders presenting with hyperuricemia or gout).
  (3) Insufficient clinical details (missing timelines of symptom progression).
  (4) Non-original research (reviews, editorials, conference abstracts).

### 3.3. Study Selection

Following the removal of duplicates, two reviewers (M.K. and K.K.) independently screened titles and abstracts, followed by full-text evaluation, using the Rayyan [18] systematic review platform. Disagreements were resolved by a third reviewer (B.R.).

### 3.4. Data Extraction

Data were extracted by M.K. and K.K., with any discrepancies resolved by a third author (B.R.). Collected variables included demographic data, *PRPS1* variant, reported diagnosis, family history, age at hearing loss onset, age at neurological onset, age at ophthalmological onset, and presence of neurological, ophthalmological, and musculoskeletal manifestations, as well as immune involvement and treatment. Variables not reported in the source publication were recorded as NR (not reported) and excluded from the corresponding analyses.

### 3.5. Data Synthesis and Statistical Analysis

The primary outcome was the symptomatic delay—defined as the interval between the onset of sensorineural hearing loss and the onset of neurological manifestations. Additional outcomes comprised neurological, ophthalmological, and musculoskeletal findings, audiological status, immune involvement, and treatment response. To characterize the age-dependent accumulation of symptoms, Kaplan–Meier survival curves were constructed for hearing loss onset and neurological onset separately, treating age at symptom onset as the time variable and the occurrence of the respective symptom as the event. Furthermore, descriptive statistics—including the median, 95% confidence intervals (CIs), and range—were calculated for four key clinical variables: the symptomatic delay, the age of onset for hearing loss, neurological manifestations, and ophthalmological signs. The methodological quality of the included case reports was assessed using the Joanna Briggs Institute (JBI) Critical Appraisal Tool for Case Reports [19]. All analyses and visualizations were performed in Python (version 3.12) [20] and Biorender.com. Gemini 3.1 Pro (Google LLC, Mountain View, CA, USA) provided stylistic editing assistance during manuscript preparation. The authors reviewed all AI-assisted content and take full responsibility for this publication.

## 4. Results

### 4.1. Summary of Included Studies

**Figure 2:**
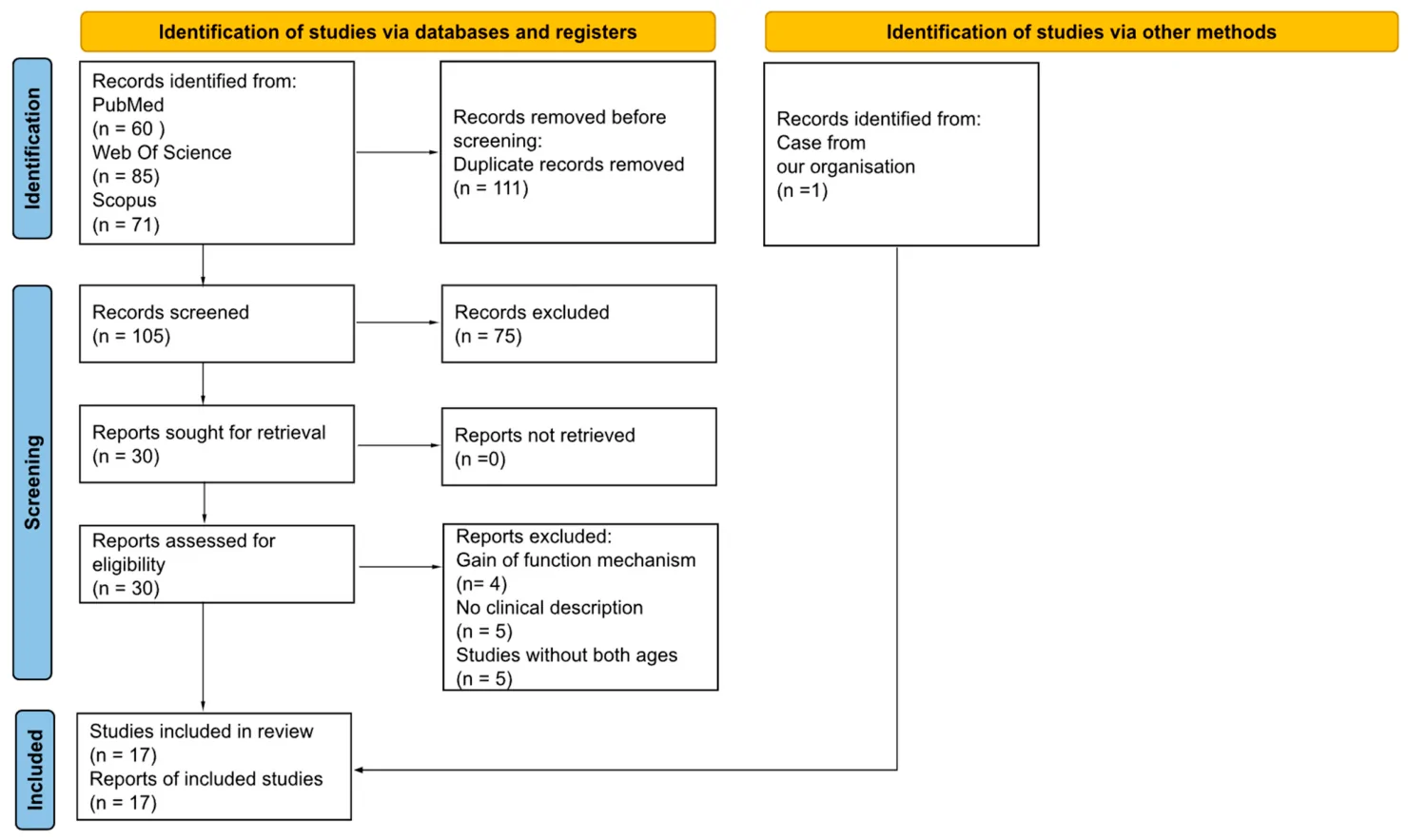
PRISMA protocol for article selection.

The database search retrieved 216 records (PubMed n = 60, Web of Science n = 85, Scopus n = 71). After the removal of 111 duplicates, 105 titles and abstracts were screened, of which 75 were excluded. Of the 30 reports assessed for full-text eligibility [7,9,14,21,22,23,24,25,26,27,28,29,30,31,32,33,34,35,36,37,38,39,40,41,42,43,44,45,46], 14 were excluded: four described gain-of-function *PRPS1* variants [35,36,37,38], five lacked sufficient clinical description [5,43,44,45,46], and five did not provide onset ages for both core symptoms [9,39,40,41,42]. A detailed table of these excluded studies, encompassing a total of 20 individual patients, is provided in Supplementary Material Table S1. A total of 17 studies, including our index case, yielded a final cohort of 19 patients (Figure 2).

### 4.2. Characteristics of Included Studies

The clinical and genetic characteristics of all included individuals are summarized in Table 1. The cohort comprised predominantly male patients (74%), representing multiple ethnicities across 5 continents. Notably, 53% of cases had a positive family history consistent with X-linked inheritance, 42% were sporadic or de novo, and 1 case (5%) had an unreported family history. The majority of patients were classified as CMTX5 or Arts syndrome, with a subset presenting an overlapping phenotype. A variety of distinct pathogenic *PRPS1* variants were identified, the majority being private mutations reported in single families. Peripheral neuropathy, areflexia, and motor deficits were universal findings; ophthalmological involvement and immune dysfunction were present in a substantial proportion of cases, though inconsistently reported across studies. The methodological quality of the included studies was generally moderate, with a mean score of 5.7/8. The most frequent reporting deficiencies concerned therapeutic protocols (Q5), post-intervention outcomes (Q6), and adverse events (Q7) (see Table S2 for detailed assessment).

### 4.3. Audiological Findings

**Figure 3:**
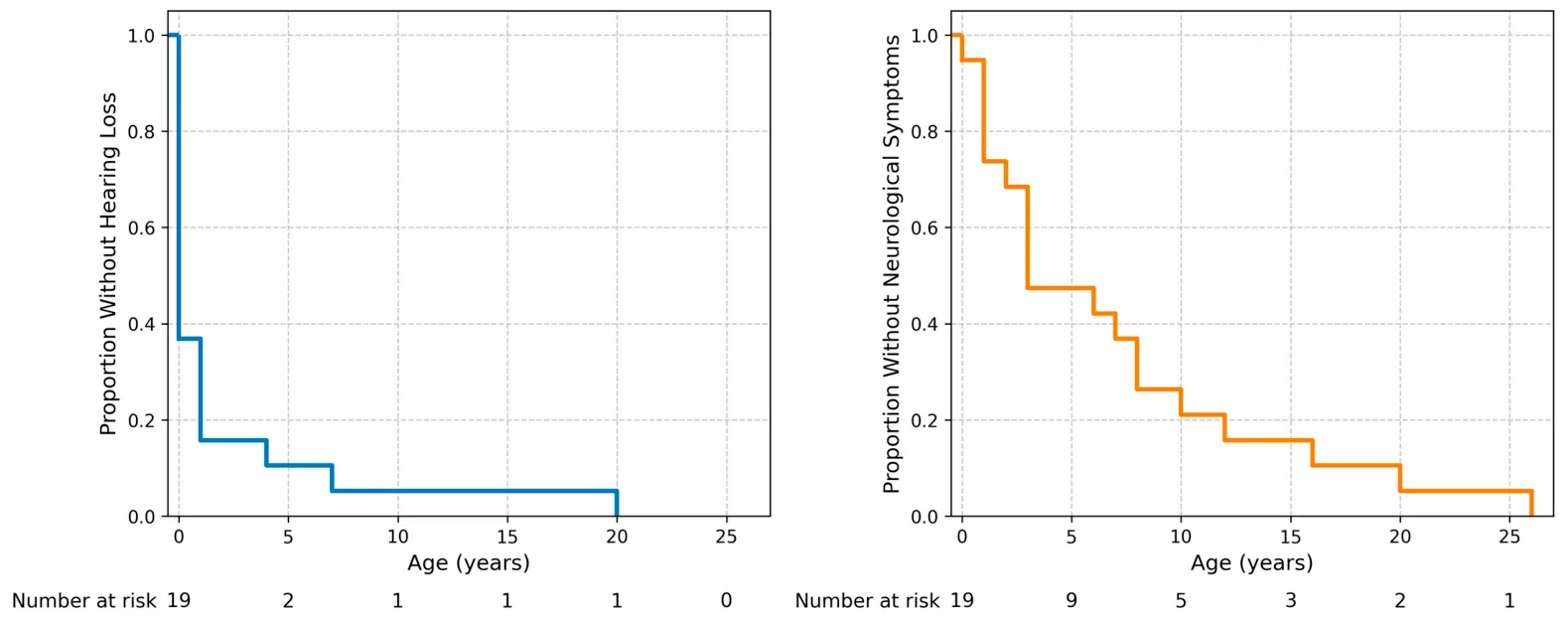
Kaplan–Meier curves showing age at onset of the core symptoms of PRPP synthetase deficiency. (**Left**): hearing loss; (**Right**): polyneuropathy.

Sensorineural hearing loss onset occurred at a median age of 0 years (95% CI: 0–1, range 0–20 years). Twelve patients (63%) had congenital hearing loss; a further four (21%) were diagnosed at 1 year of age. When stratified by sex, the median onset for males (n=14) was 0 years, whereas females (n=5) presented slightly later with a median of 1 year. The Kaplan–Meier curve for time to hearing loss onset is presented in Figure 3 (left panel).

### 4.4. Neurological Findings and Symptomatic Delay

Neurological onset occurred at a median age of 3 years (95% CI: 1–8, range 0–26 years); see Figure 3 (right panel). Stratification by sex revealed that males developed neurological manifestations earlier (median 3 years) compared to females (median 7 years). The symptomatic delay—defined as the interval between hearing loss onset and first neurological manifestation—was a median of 3 years (95% CI: 1–7, range 0–19 years). The longest individual delay in the cohort was 19 years, observed in the female patient described by Uner et al. (2025) [27]; the case presented here exhibited the second longest delay at 15 years. Two patients (11%) had concurrent onset of both symptoms (delay = 0).

### 4.5. Ophthalmological Findings

For the subset of patients with available age-of-onset data for ophthalmological manifestations (n = 10), the median age of manifestation was 11.5 years (95% CI: 0–25; range: 0–33 years). This subset was too small (n = 10) for Kaplan–Meier analysis.

### 4.6. Therapeutic Interventions and Clinical Outcomes

Within the reviewed cohort, S-adenosylmethionine (SAM) and/or nicotinamide riboside (NR) supplementation was attempted in a subset of patients [23,29,30,32]. Due to the lack of controlled trials, varying disease stages at treatment initiation, and a reliance on subjective parental or patient reports, the true clinical efficacy of these interventions remains difficult to definitively establish. Available case data suggest that while metabolic supplementation may potentially stabilize biochemical profiles, reduce the frequency of infections, and subjectively improve fatigue or motor function in some individuals [23,29,30], it does not appear to reverse established structural neurological or ophthalmological damage [29,32]. In our index case, the administration of SAM (250 mg/day) at age 16 did not lead to a clinically observable reversal of peripheral neuropathy or optic atrophy.

## 5. Discussion

### 5.1. Temporal Dissociation and Differential Tissue Vulnerability

The diagnostic latencies observed within the cohort of PRPP synthetase deficiency individuals highlight a suggested temporal dissociation in tissue-specific vulnerability to phosphoribosyl pyrophosphate (PRPP) depletion [6,32]. The auditory apparatus appears to exhibit acute, early susceptibility, typically presenting as congenital or pre-lingual sensorineural hearing loss. This may not merely be a consequence of “high metabolic demand,” but could specifically reflect the dependence of the stria vascularis on a continuous ATP supply for Na^+^/K^+^-ATPase pumps to maintain the endocochlear potential, alongside the high sensitivity of hair cells to intracellular GTP depletion [9,12,47]. Conversely, the central and peripheral nervous systems may demonstrate prolonged cellular redundancy prior to the onset of progressive neurodegeneration [6]. The median symptomatic delay of 3 years (95% CI: 1–7) between auditory and motor deficits may illustrate the motor system’s significant capacity to temporarily buffer this energetic crisis. This transient resilience likely stems from a highly efficient initial compensation via purine salvage pathways (HGPRT/APRT) or tissue-specific expression of the PRPS2 paralog in motor neurons, which may eventually be exhausted, leading to axonal homeostasis breakdown [48]. Furthermore, these results indicate that visual impairment generally develops significantly later (median 11.5 years) than both sensorineural hearing loss (median 0 years) and initial neurological deficits (median 3 years). This underscores an even more prolonged period of potential compensation in the optic nerve and retina, suggesting a distinct gradient of tissue vulnerability across the sensory and motor systems [6,32].

### 5.2. Beyond Rigid Syndromic Boundaries

Our data suggest limitations in rigid syndromic categorization of patients to one of the disease entities resulting from the germline loss-of-function variants in *PRPS1*. The consistent presence of peripheral neuropathy and motor deficits, coupled with the variable penetrance of optic atrophy and immunodeficiency, supports the concept of a continuous spectrum of enzymatic insufficiency rather than discrete clinical entities like DFNX1, CMTX5, and Arts syndrome [32,39]. Furthermore, the marked phenotypic heterogeneity—even among recurrent variants in unrelated families, as illustrated by our case—indicates the presence of specific disease modifiers [39]. As illustrated by the conceptual model, the position of a patient along this phenotypic continuum is dictated not only by the primary PRPS1 residual activity but also by a network of secondary modifiers. This is particularly evident in heterozygous females, where the pattern of X-chromosome inactivation (XCI) acts as a critical phenotype modifier. Depending on whether XCI is favorably skewed or random, females can experience a delayed onset of symptoms compared to hemizygous males—as reflected by the delayed median onset of neurological signs in our female cohort (7 years vs. 3 years in males). In addition to XCI and DNA methylation patterns, the severity is further modulated by polymorphisms dictating the efficiency of salvage pathway enzymes (HGPRT, APRT) and individual variances in intracellular NAD+ turnover kinetics [6,23]. Reconceptualizing these conditions as a singular *PRPS1*-related neurometabolic continuum could provide a more robust pathobiological framework and suggests the potential benefit of unifying clinical management protocols [32].

Crucially, the clinical trajectory of *PRPS1* variants with a pre-assumed loss of function is highly heterogeneous and does not universally result in severe neurodegeneration. Some patients exhibit a stable clinical plateau, where the disease remains confined to its initial manifestation. For example, several excluded reports of DFNX1 describe individuals with early-onset hearing loss who did not develop subsequent motor deficits (cases reported by Liu et al., Gandía et al., and Kim et al.) [5,9,41]. This phenotypic diversity is further highlighted by atypical presentations, such as isolated retinal dystrophies lacking auditory signs (Fiorentino et al.) [42] or early motor impairment without sensorineural hearing loss (Puusepp et al.) [40]. Therefore, disease progression appears non-linear and is likely shaped by the specific variant and its residual enzymatic activity.

### 5.3. Metabolic Rescue and the Window of Reversibility

**Figure 4:**
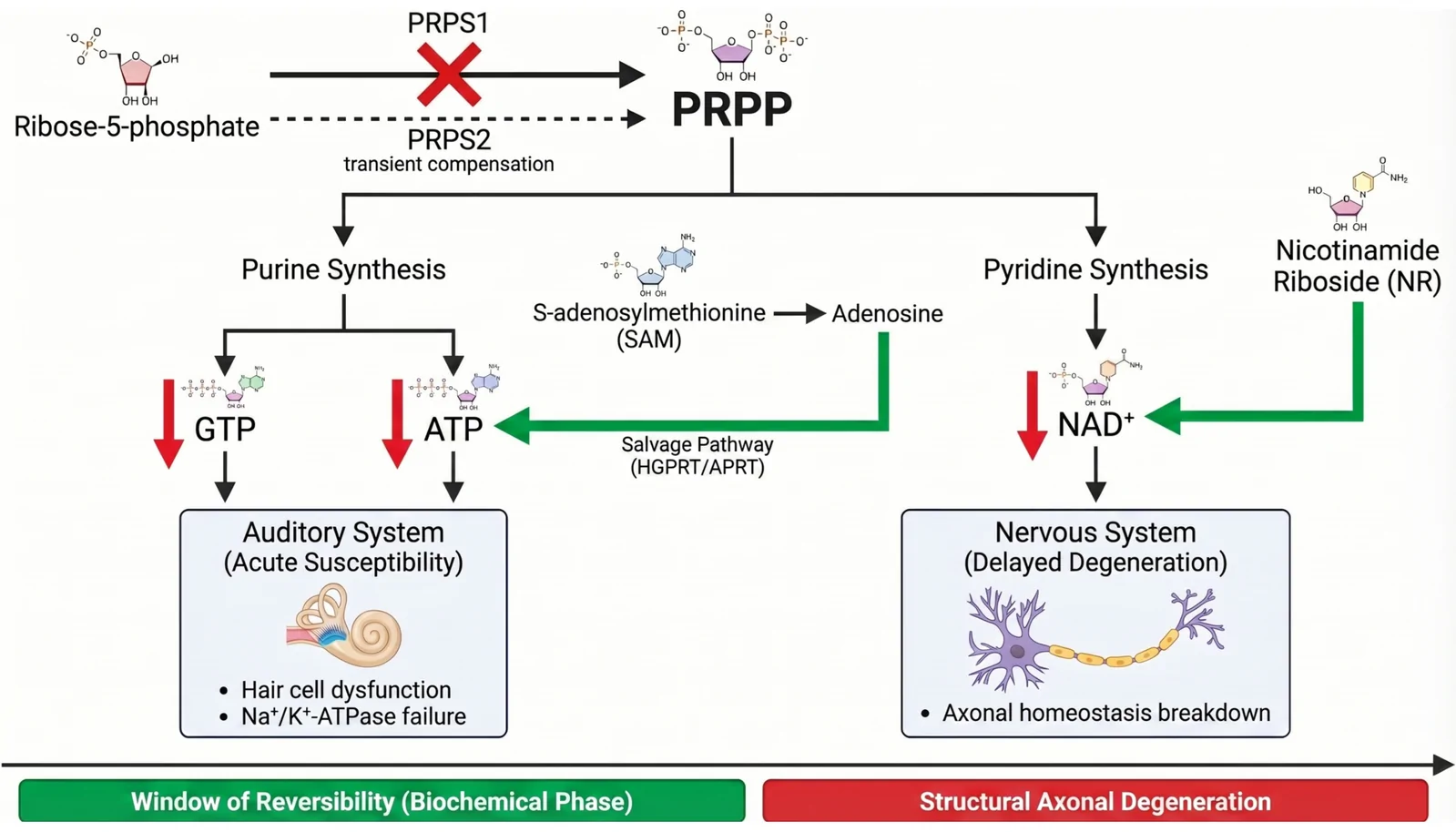
Pathomechanism of PRPP deficiency. Created in BioRender. Rodziewicz, B. (2026) https://BioRender.com/mgyhsww (access on 28 June 2026).

Therapeutic interventions utilizing S-adenosylmethionine (SAM) and nicotinamide riboside (NR) may delineate the biological limits of plasticity in *PRPS1*-related neurodegeneration [23]. These compounds may not merely act as generic “purine replacements”; rather, they are thought to precisely bypass PRPP-dependent biosynthetic pathways. Nicotinamide riboside appears to act as a direct precursor, bypassing the enzymatic block, potentially rescuing the cellular NAD+ pool [23]. Mechanistically, nicotinamide riboside enters the NAD+ pool through phosphorylation by nicotinamide riboside kinases to nicotinamide mononucleotide and onward to NAD+, a route that is independent of PRPP and thus circumvents the deficient NAPRT/NAMPT reactions, whereas the adenine liberated upon demethylation of SAM is recaptured by APRT to regenerate adenine nucleotides—together explaining how these agents can restore NAD(P) and ATP/GTP pools without correcting the primary enzymatic lesion [6,23]. SAM is proposed to serve primarily as a methyl donor (correcting secondary hypomethylation) and, following demethylation, may provide an intracellular source of adenosine to replenish ATP pools via the salvage pathway [6]. However, the available clinical data suggest a limitation to this metabolic rescue. While SAM and NR supplementation might offer some systemic stabilization or subjective improvement in fatigue and infection susceptibility [23,29,30], these interventions appear insufficient to reverse established neuromuscular or retinal damage once structural degeneration has occurred [29,32]. This suggests a potential pathogenetic threshold: once the energetic deficit initiates secondary structural axonal degeneration, the disease process may uncouple from the initial biochemical trigger. The variance in SAM dosing (20 to 1600 mg daily) likely reflects empirical clinical attempts to overcome blood–brain barrier (BBB) penetration limits as the disease progresses structurally. Consequently, the therapeutic window for metabolic rescue appears to be confined to the purely biochemical phase—prior to the structural collapse of nerve fibers [6,48]. The proposed pathomechanism and the rationale for this metabolic rescue are visually presented in Figure 4.

### 5.4. Molecular Architecture of PRPS1 and the Structural Basis of the Loss-of-Function Continuum

**Figure 5:**
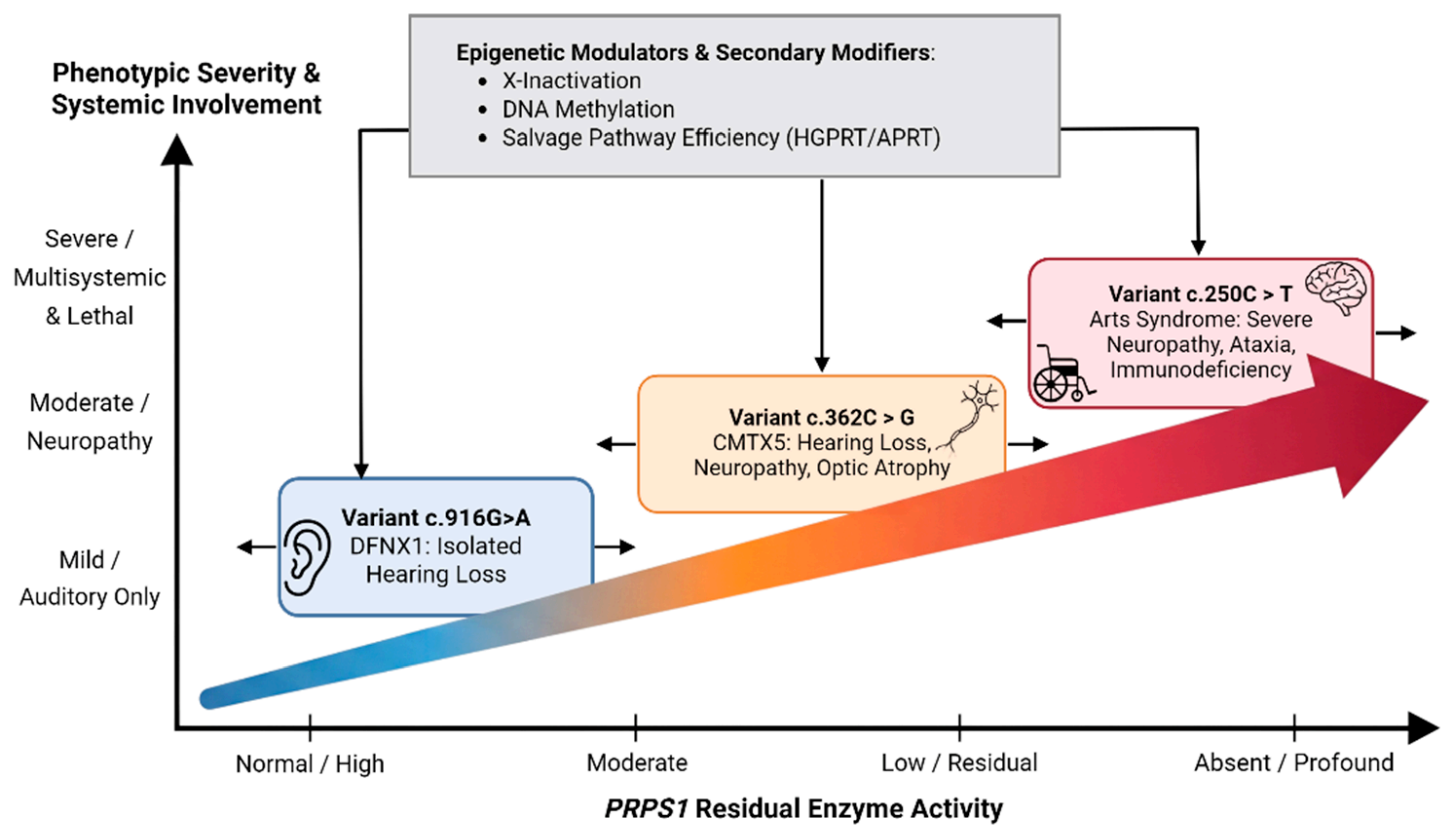
Epigenetic modulators of PRPS1 enzyme activity. Created in BioRender. Rodziewicz, B. (2026) https://BioRender.com/dsk0yvl (access on 28 June 2026).

The clinical continuum described above has a defined structural counterpart at the level of the PRPP synthetase 1 enzyme. Each protomer contributes a β2–3 ATP-binding motif, a β9–10 ribose-5-phosphate-binding motif, and a flexible catalytic loop. Additionally, a “flag” region donated by the adjacent subunit completes the ATP-binding cleft [10,49]. Three catalytic dimers then associate to form the functional hexamer. Within this complex, each regulatory site is built from residues contributed by three adjacent protomers, accommodating either the activator (phosphate) or the inhibitor (ADP) [50]. Pathogenic loss-of-function substitutions are distributed across all of these functional elements—the substrate-binding motifs, the catalytic loop, and the dimer and hexamer interfaces. They reduce activity through distinct mechanisms, including impaired ATP or ribose-5-phosphate binding, defective catalysis, and destabilized folding or oligomerization [12,49]. Because each mechanism lowers catalytic output to a different extent, the resulting residual PRPS1 activity directly dictates the clinical severity, as mapped in Figure 5. Variants with relatively preserved function (e.g., c.916G>A) typically cause isolated hearing loss. A further drop in activity, seen with our c.362C>G variant, may expand the phenotype to include neuropathy and optic atrophy. Finally, nearly absent enzyme function (e.g., c.250C>T) may lead to the severe, multisystemic Arts syndrome [6,39]. In heterozygous females, the effective activity—and therefore disease severity—is further modulated by the pattern of X-chromosome inactivation [12].

The variant identified in our case, c.362C>G (p.Ala121Gly), substitutes a small, highly conserved alanine with glycine. In silico tools suggest a potentially deleterious impact: SIFT [51] scores it as deleterious (0.00), PolyPhen-2 [52] as damaging (0.624), ClinVar [15] (ID: 100767) as pathogenic, and AlphaMissense classifies it as likely pathogenic with a pathogenicity score of 0.687 [53]. AlphaFold modeling [54] (UniProt: P60891; pLDDT = 94.81) indicates that Ala121 resides within a conserved structural element. Replacing the native alanine with glycine may introduce localized backbone flexibility, potentially imposing an entropic penalty that could destabilize motifs involved in hexamer oligomerization or allosteric control. Pending direct functional validation, these predicted structural alterations align with known PRPS1 loss-of-function mechanisms, where impaired oligomeric stability is thought to reduce enzymatic output. Notably, the marked interfamilial variability observed for this recurrent substitution cannot be attributed to the primary mutation alone. The earlier, relatively uniform motor onset in the previously reported Korean family contrasts with the later onset and consistent ocular involvement in our family. As previously discussed, this discrepancy implicates secondary modifiers of residual nucleotide flux, such as inter-individual differences in salvage pathway efficiency and NAD+ turnover, superimposed on an identical molecular lesion [6,39]. This reinforces the interpretation of *PRPS1*-related disease as a quantitative, activity-dependent continuum rather than a set of discrete Mendelian entities.

### 5.5. Intracellular Molecular Pathology: Purinosome Dynamics and NAD+ Depletion

Beyond the immediate disruption of purine nucleotide (ATP/GTP) synthesis, the molecular pathology of *PRPS1* variants appears to be driven by complex intracellular cascades. Evidence indicates that de novo purine biosynthesis operates within coordinated multienzyme complexes known as purinosomes, whose assembly and functional integrity are dynamically regulated by molecular chaperones, particularly HSP90 [55]. The impaired hexameric assembly documented for several disease-associated *PRPS1* variants [50] could initiate a downstream cascade that lowers catalytic output and may hypothetically extend to the assembly of purinosomes, provoking secondary proteostatic stress. Concurrently, PRPP is the obligate substrate for the phosphoribosyltransferases that regenerate NAD+—nicotinamide phosphoribosyltransferase (NAMPT) in the salvage route and nicotinate phosphoribosyltransferase (NAPRT) in the Preiss–Handler route—while the de novo route from tryptophan is likewise PRPP-dependent at its terminal step [6]. Consequently, *PRPS1* dysfunction is expected to reduce the intracellular NAD+ pool [6,23]. In highly polarized and metabolically demanding cells, such as peripheral motor neurons, this NAD+ deficit possibly compromises mitochondrial oxidative phosphorylation and sirtuin-mediated cellular stress responses. Impaired purinosome-dependent purine supply, chronic bioenergetic starvation, and a decline in NAD+-dependent axon-maintenance mechanisms may together contribute to the delayed, structural axonal degeneration observed in these patients [48,56]. However, while compelling, the precise impact of PRPS1 hexamer destabilization on global purinosome integrity remains speculative and warrants future functional validation. This intricate pathomechanism underscores the necessity for early, targeted metabolic interventions aimed at restoring these molecular networks prior to irreversible structural collapse [6,23].

### 5.6. Interfamilial Heterogeneity

Differences were also observed regarding the onset of motor symptoms. In the Korean family, gait disturbance was reported to begin between 4 and 6 years of age, whereas in our Polish family, motor symptoms were documented to begin between 14 and 15 years of age in three affected individuals. For two additional affected males, the age at onset could not be reliably determined, and one individual remains presymptomatic for peripheral neuropathy. Notably, clinical presentation appeared relatively homogeneous within each family but differed substantially between families carrying the same variant. Taken together, these findings suggest the presence of marked interfamilial phenotypic variability, particularly with respect to the age at onset of motor symptoms and the occurrence of ocular manifestations.

### 5.7. Limitations

This review is inherently constrained by the ultra-rare nature of *PRPS1*-related disorders. Relying exclusively on 19 cases derived from case reports and small series precludes formal meta-analysis and introduces unavoidable reporting heterogeneity. For example, the lack of standardized follow-up protocols across different studies means that the depth of clinical phenotyping—such as the timing of neurophysiological assessments or ophthalmological examinations—varies significantly among the reported patients.

To mitigate these limitations, we included only genetically confirmed cases with precise, individual-level clinical timelines. While this stringent approach ensures the reliability of findings regarding the temporal dissociation of symptoms, it reduces the sample size. Nevertheless, this restricted evidence base limits the systematic evaluation of metabolic interventions. The available data lack controlled therapeutic trials, and the reported use of interventions, such as S-adenosylmethionine (SAM), involves widely divergent dosages and differing stages of disease progression at the time of initiation, preventing definitive conclusions about their efficacy. Another significant limitation of this study is the restricted sample size, which necessitated combining male and female patients in the Kaplan–Meier analyses. As our data indicate, the disease course in heterozygous females differs from that in hemizygous males (median onset). This phenotypic variability, driven by X-chromosome inactivation, introduces confounding effects that may distort the overall survival analysis.

Furthermore, inherent publication bias likely overrepresents severe or rapidly progressive phenotypes, suggesting that milder or late-onset presentations remain systematically underreported. It is highly probable that patients with atypical or less severe clinical trajectories are frequently misdiagnosed with idiopathic polyneuropathies or isolated non-syndromic hearing loss, leaving a significant portion of the disease spectrum unrecognized.

Finally, a notable methodological limitation of our analysis is the requirement for precise onset data for both auditory and neurological symptoms to calculate diagnostic latency. Consequently, patients with stable, non-progressing phenotypes or atypical presentations lacking one of the core features were excluded from the primary cohort. This inclusion criterion naturally leads to the overrepresentation of progressive cases. Thus, the median diagnostic latencies reported here should be interpreted with caution, as the natural history of *PRPS1* deficiency is highly heterogeneous, ranging from strictly isolated sensory defects to progressive multisystemic disorders.

## 6. Conclusions

In conclusion, our findings suggest that *PRPS1* loss-of-function variants may represent a continuous neurometabolic spectrum rather than strictly distinct clinical syndromes (DFNX1, CMTX5, and Arts syndrome). The disease frequently follows a characteristic timeline: acute, isolated hearing loss typically occurs early in life, whereas progressive structural neurodegeneration tends to develop years later. This time gap in some patients between symptoms is a major clinical finding in our study. However, given the marked phenotypic heterogeneity within this patient group, it is crucial to recognize that not all individuals will progress along this entire continuum, and individualized disease trajectories must be carefully taken into account. Because existing metabolic treatments (such as SAM or nicotinamide riboside) appear to have limited efficacy once irreversible physical nerve damage has occurred, this early diagnostic phase might represent a crucial therapeutic window. Therefore, it may be beneficial for clinicians to consider *PRPS1* deficiency in cases of unexplained early-onset deafness. Recognizing this sequence of events could potentially allow for the earlier initiation of metabolic rescue therapies before severe motor decline begins.

## Figures and Tables

**Table 1:** Summary of clinical and genetic characteristics of reported patients with *PRPP synthetase deficiency*-related disorders.

No	Author & Year	Ethnicity	Sex	*PRPS1* Variant (cDNA)	Clinical Phenotype	Family History	Age at Hearing Loss Onset (Years)	Age at Neurological Onset (Years)	Symptomatic Delay (Years)	Age at Ophthalmologic Signs	Neuropathy / Areflexia	Motor / Gait Deficits	Ophthalmologic Signs	Immune Issues	Therapies
1	Shirakawa et al., 2022 [21]	Japanese (Asian)	M	c.82G>C	CMTX5	Sporadic (de novo)	0	3	3	11	+	+	+	-	NR
2	Lerat et al., 2019 [22]	French (Caucasian)	M	c.202A>T	CMTX5	Familial (mother carrier)	1	8	7	NR	+	+	+	-	NR
3	Lenherr et al., 2021 [23]	Swiss (Caucasian)	M	c.250C>T	Arts	Sporadic (de novo)	0	1	1	1	+	+	+	+	SAM (400 mg/day); Nicotinamide riboside (300 mg/day)
4	Alzahem et al., 2024 [24]	Saudi (Arab)	F	c.287G>A	CMTX5	Sporadic (de novo)	20	26	6	33	+	+	+	NR	NR
5	Nishikura et al., 2019 [25]	Japanese (Asian)	M	c.319A>G	CMTX5	Familial (2 brothers)	0	8	8	-	+	+	-	-	NR
6	Nishikura et al., 2019 [25]	Japanese (Asian)	M	c.319A>G	CMTX5	Familial (2 brothers)	1	2	1	-	+	+	-	-	NR
7	Meng et al., 2019 [26]	Chinese (Asian)	M	c.334G>C	CMTX5	Familial (mother carrier)	0	3	3	NR	+	+	+	-	NR
8	Uner et al., 2025 [27]	Brazilian (Hispanic)	F	c.359G>T	Arts	Sporadic (de novo)	1	20	19	13	+	+	+	NR	NR
9	Our case	Polish	M	c.362C>G	CMTX5 / Arts	Familial (5 maternal relatives)	1	16	15	19	+	+	+	-	SAM (250 mg/day), Cochlear implant
10	Park et al., 2013 [14]	Korean (Asian)	M	c.362C>G	CMTX5	Familial (2 maternal uncles)	0	6	6	-	+	+	-	-	Cochlear implant
11	Maruyama et al., 2016 [28]	Japanese (Asian)	M	c.367C>G	Arts	Familial (3 maternal uncles)	0	1	1	-	+	+	-	-	NR
12	Lee et al., 2023 [29]	American (Caucasian)	M	c.383A>T	Arts	Familial (mother & aunt)	0	0	0	NR	+	+	+	-	SAM (20–40 mg/day); Nicotinamide riboside (30 mg/day); Cochlear implant
13	Prasun et al., 2025 [30]	American (NR)	M	c.383A>T	NR	NR	4	10	6	1	+	+	+	NR	SAM (1600 mg/day); Nicotinamide riboside (800 mg/day)
14	Zhou et al., 2021 [31]	Chinese (Asian)	M	c.421C>T	Arts	Sporadic (de novo)	0	1	1	-	+	+	-	-	SAM
15	Stajer et al., 2023 [32]	Slovenian (Caucasian)	M	c.424G>A	CMTX5 / Arts	Familial (mother & sister carriers)	0	1	1	0	+	+	+	+	SAM (500 mg/day)
16	Sather et al., 2024 [33]	American (Caucasian)	F	c.506C>T	CMTX5 / Arts	Sporadic (de novo)	0	3	3	NR	+	+	+	-	NR
17	Zocche et al., 2026 [34]	British (Caucasian)	F	c.640C>T	CMTX5	Sporadic	0	3	3	0	+	+	+	NR	NR
18	Synofzik et al., 2014 [7]	German (Caucasian)	M	c.830A>C	CMTX5 / Arts	Familial (sister)	0	12	12	12	+	+	+	+	NR
19	Uner et al., 2025 [27]	Australian (Caucasian)	F	c.864+1G>T	Arts	Sporadic (de novo)	7	7	0	25	+	+	+	NR	NR

Abbreviations: M = male; F = female; NR = not reported; SAM = S-adenosylmethionine. Symbols: (+) = present; (-) = absent. Notes: *Sporadic* indicates cases in which the mutation was confirmed as de novo, or there is no known family history. *Familial* indicates a positive family history of the disease or confirmed carrier status in relatives. Grey shading indicates our index case.

## Data Availability

All data underlying the results are available in Table 1. Clinical data for the index case are detailed in the Section 2. The remaining dataset was derived from previously published, publicly available literature.

## Supplementary Materials

The following supporting information can be downloaded at https://www.mdpi.com/article/10.3390/biom16081164/s1, Table S1. Excluded studies; Table S2: JBI Critical Appraisal of the Included Case Reports; File S1: PRISMA checklist.

## Abbreviations

The following abbreviations are used in this manuscript:

*PRPS1*	Phosphoribosyl pyrophosphate synthetase 1
PRPP	Phosphoribosyl pyrophosphate
CMTX5	Charcot–Marie–Tooth disease type X5
DFNX1	X-linked non-syndromic hearing loss
HMSN	Hereditary motor and sensory neuropathy
SAM	S-adenosylmethionine
NGS	Next-generation sequencing
BBB	Blood–brain barrier
NAD+	Nicotinamide adenine dinucleotide
NR	Nicotinamide riboside
pLDDT	Predicted local distance difference test
