# Supplementary material for: Age-Dependent Progression of Neurological Involvement in PRPP Deficiency: Insights from a Four-Generation Family and Systematic Review
Source: Biomolecules. 2026 Aug 11;16(8):1164. doi: 10.3390/biom16081164 (PMC13511699; doi:10.3390/biom16081164)
Supplement: Supplementary file 1 [file biomolecules-16-01164-s001.zip › Table S1.pdf]

TABLE S1 – EXCLUDED STUDIES

No	Author & Year	Ethnicity	Sex	*PRPS1* Variant (cDNA)	Clinical Phenotype	Family History	Age at Hearing Loss	Age at Neurological Onset (years)	Symptomatic delay (years)	Age at Ophthalmologic signs	Neuropathy / Areflexia	Motor / Gait Deficits	Ophthalmologic Signs	Immune Issues	Reason for exclusion
1	Porrmann et al. [35]	German	M	c.573G>C	hyperuricemia	Familial (mother carrier)	-	NR	-	NR	-	-	-	-	Superactivity
2	Moran et al. [36]	NR	M	c.424G>C	Hyperuricemia / Arts syndrome spectrum	Familial (mother carrier)	0	0	0	NR	+	+	+	+	Superactivity
3	Chen et al. [37]	NR	NR	c.154G>C	Hyperuricemia	Familial	NR	NR	-	NR	NR	NR	NR	NR	Superactivity

TABLE S1 – EXCLUDED STUDIES

4	Becker et al. [38]	NR	M	c.385C>A	Hyperuricemia	Familial	NR	NR	-	NR	NR	NR	NR	NR	Superactivity
5	Robusto et al. [39]	Italian	M	c.343A>G	CMTX5 spectrum	Familial (mother carrier)	NR	NR	-	-	-	+	-	-	Lack of both ages
6	Puusepp et al. [40]	Estonian	M	c.130A>G	Atypical Arts syndrome	Familial (mother & grandmother carriers)	-	0	-	-	+	+	-	+	Lack of both ages
7	Kim et al. [41]	Korean	M	c.244G>C	DFNX1	Familial (mother carrier)	3	-	-	-	-	-	-	-	Lack of both ages

TABLE S1 – EXCLUDED STUDIES

8	Liu et al. [9]	Chinese	M	c.193G>A	DFNX1	Familial (mother carrier)	5	-	-	-	-	-	-	-	Lack of both ages
9	Liu et al. [9]	Chinese	M	c.869T>C	DFNX1	Familial (mother carrier)	NR	-	-	-	-	-	-	-	Lack of both ages
10	Liu et al. [9]	British	M	c.259G>A	DFNX1	Familial (mother carrier)	1	-	-	-	-	-	-	-	Lack of both ages
11	Liu et al. [9]	America n	M	c.916G>A	DFNX1	Familial (mother carrier)	7	-	-	-	-	-	-	-	Lack of both ages

TABLE S1 – EXCLUDED STUDIES

12	Fiorentino et al. [42]	British	F	c.47C>T	Retinal Dystrophy	Familial (affected females only)	-	-	-	19	-	-	+	-	Lack of both ages
13	Fiorentino et al. [42]	British	F	c.641G>C	Cone-rod dystrophy	Familial (affected females only)	-	-	-	26	-	-	+	-	Lack of both ages
14	Gandía et al. [5]	Spanish	M	c.824T>C	DFNX1	Familial	0	-	-	-	-	-	-	-	Lack of both ages
15	Hughes et al. [43]	NR	M	NR	CMTX5	NR	0	NR	-	NR	+	+	-	-	lack of clinical description

TABLE S1 – EXCLUDED STUDIES

16	Georgiou et al. [44]	NR	F	c.47C>T	Sector Retinitis Pigmentosa	Familial	NR	NR	-	45	NR	NR	+	NR	lack of clinical description
17	Tinker et al. [45]	America n	F	c.307-2A>G	Microcornea & Glaucoma	Sporadic (de novo)	NR	NR	-	4	NR	NR	+	NR	lack of clinical description
18	Gandía et al. [5]	Spanish	M	c.917G>A	Arts syndrome spectrum	Familial	1	NR	-	-	NR	+	-	-	lack of clinical description
19	Gandía et al. [5]	Spanish	M	c.917G>A	DFNX1 + structural foot anomaly	Familial	1	NR	-	-	NR	+	-	-	lack of clinical description

TABLE S1 – EXCLUDED STUDIES

20	Buianova et al. [46]	Russian	M	c.202A>G	CMTX5	Familial (mother carrier)	NR	NR	-	NR	NR	NR	NR	NR	lack of clinical description
