# Supplementary material for: Age-Dependent Progression of Neurological Involvement in PRPP Deficiency: Insights from a Four-Generation Family and Systematic Review
Source: Biomolecules. 2026 Aug 11;16(8):1164. doi: 10.3390/biom16081164 (PMC13511699; doi:10.3390/biom16081164)
Supplement: Supplementary file 1 [file biomolecules-16-01164-s001.zip › Table S2-JBI Critical Appraisal of the Included Case Reports.pdf]

TABLE S2: JBI Critical Appraisal of the Included Case Reports

Author & Year	Q1	Q2	Q3	Q4	Q5	Q6	Q7	Q8	Score
Shirakawa et al. (2022) [21]	Y	Y	Y	Y	N	N	N	Y	5/8
Lerat et al. (2019) [22]	Y	Y	Y	Y	N	N	N	Y	5/8
Lenherr et al. (2021) [23]	Y	Y	Y	Y	Y	Y	N	Y	7/8
Alzahem et al. (2024) [24]	Y	Y	Y	Y	N	N	N	Y	5/8
Nishikura et al. (2019) P1 [25]	Y	Y	Y	Y	N	N	N	Y	5/8
Nishikura et al. (2019) P2 [25]	Y	Y	Y	Y	N	N	N	Y	5/8
Meng et al. (2019) [26]	Y	Y	Y	Y	N	N	N	Y	5/8
Uner et al. (2025) P1 [27]	Y	Y	Y	Y	N	N	N	Y	5/8
Our case (2026)	Y	Y	Y	Y	Y	Y	Y	Y	8/8
Park et al. (2013) [14]	Y	Y	Y	Y	Y	U	N	Y	6/8
Maruyama et al. (2016) [28]	Y	Y	Y	Y	N	N	N	Y	5/8
Lee et al. (2023) [29]	Y	Y	Y	Y	Y	Y	Y	Y	8/8
Prasun et al. (2025) [30]	U	Y	Y	Y	Y	Y	N	Y	6/8
Zhou et al. (2021) [31]	Y	Y	Y	Y	Y	Y	N	Y	7/8
Stajer et al. (2023) [32]	Y	Y	Y	Y	Y	Y	N	Y	7/8
Sather et al. (2024) [33]	Y	Y	Y	Y	N	N	N	Y	5/8
Zocche et al. (2026) [34]	Y	Y	Y	Y	N	N	N	Y	5/8
Synofzik et al. (2014) [7]	Y	Y	Y	Y	N	N	N	Y	5/8
Uner et al. (2025) P2 [27]	Y	Y	Y	Y	N	N	N	Y	5/8

Q1—demographic characteristics; Q2—patient history/timeline; Q3—current clinical condition; Q4—diagnostic tests and results; Q5—interventions/treatment; Q6—post-intervention condition; Q7—adverse events; Q8—takeaway lessons. Y = Yes; N = No; U = Unclear.
